# Evaluating periportal fibrosis in schistosomiasis mansoni using the ELF [Enhanced Liver Fibrosis] score: A pilot study

**DOI:** 10.1371/journal.pntd.0014775

**Published:** 2026-10-09

**Authors:** Rebecca Dantas Thorp, Andrea Dória Batista, Carolline A. Mariz, Lilian Rose Maia Gomes de Araújo, George Tadeu Nunes Diniz, Ana Virgínia Matos Sá Barreto, Clarice Neueschwander Lins de Morais, Paula Carolina Valença Silva, Ana Lúcia Coutinho Domingues, Edmundo Pessoa Lopes

**Affiliations:** 1 Postgraduate Program in Tropical Medicine, Center of Medical Sciences, Federal University of Pernambuco [UFPE], Recife, Pernambuco, Brazil; 2 Department of Internal Medicine, Center of Medical Sciences, Federal University of Pernambuco [UFPE], Recife, Pernambuco, Brazil; 3 Department of Parasitology, Aggeu Magalhães Institute, Oswaldo Cruz Foundation, Recife, Pernambuco, Brazil; 4 Gastroenterology Division, Hospital das Clínicas/EBSERH, Federal University of Pernambuco [UFPE], Recife, Pernambuco, Brazil; 5 Department of Geoprocessing and Statistics, Aggeu Magalhães Institute, Oswaldo Cruz Foundation, Recife, Pernambuco, Brazil; 6 Maurício de Nassau University Center, Graças Campus, Recife, Pernambuco, Brazil; 7 Department of Virology, Aggeu Magalhães Institute, Oswaldo Cruz Foundation, Recife, Pernambuco, Brazil; 8 Federal University of Pernambuco [UFPE], Vitória de Santo Antão, Pernambuco, Brazil; 9 Real Hospital Português [RHP], Recife, Pernambuco, Brazil; University of Oxford, UNITED KINGDOM OF GREAT BRITAIN AND NORTHERN IRELAND

## Abstract

**Background:**

Measuring the periportal fibrosis pattern (PPF) in schistosomiasis mansoni (SM) is essential for diagnosing portal hypertension. The Enhanced Liver Fibrosis (ELF) test can be used to quantify liver fibrosis, however, few studies have been done in SM. This study evaluated serum ELF value according to the PPF pattern established by the Niamey ultrasound classification (US) and history of upper gastrointestinal bleeding (UGIB) in patients with SM.

**Methodology:**

A cross-sectional, bidirectional analytical study was carried out with patients from an endemic area and from a reference outpatient clinic for SM. A structured questionnaire was applied to collect sociodemographic and clinical data, and blood samples were collected for ELF determination. US was performed by the same examiner.

**Principal Findings:**

Among the 196 patients evaluated (58% women; mean age of 45.04 years ± 15.30 years) 18 (%) presented with Niamey US pattern A + B; 64 (%) pattern C; 56 (%) pattern D; 49 (%) pattern E and 9 (%) pattern F. The median ELF values were higher the greater the PPF intensity: 8.36 in A + B; 8.40 in C; 8.64 in D; 8.99 in E and 9.75 in F Niamey pattern (p = 0.0021). There was no difference in the mean ELF serum value between patients with and without history of UGIB. The ELF was able to differentiate patients without PPF, with mild, moderate and advanced PPF: A + B vs C vs D vs E + F (p = 0.0024), as well as patients with advanced PPF from those without fibrosis or with moderate fibrosis: A + B vs E + F (p = 0.0464) and C + D vs E + F (P = 0.0007).

**Conclusion/ /Significance:**

The ELF test demonstrated potential as a tool for assessing PPF in SM patients, as it could differentiate those with minimal fibrosis from those with moderate or advanced fibrosis.

## Author summary

Schistosomiasis is considered one of the main causes of fibrogenic liver disease and affects more than 250 million people worldwide. The diagnosis of the presence and severity of periportal fibrosis (PPF) is essential for preventing its complications, especially in patients with more advanced patterns of PPF, who may present portal hypertension and are subject to upper gastrointestinal bleeding. The liver fibrosis evaluation is through direct or indirect markers, used alone or together, known as indices. The Enhanced Liver Fibrosis (ELF) test is an index that combines three markers: hyaluronic acid, tissue inhibitory factor of metalloproteinases-1 (TIMP-1) and amino-terminal propeptide of type III procollagen (PIIINP). This index has demonstrated prognostic value in the early stages of chronic hepatitis B and C or autoimmune hepatitis and can predict clinical outcomes. In this study, the ELF was able to separate patients without PPF or with doubtful PPF from the other patterns, as well as separating those with advanced or very advanced PPF from the others with less PPF. This capacity becomes fundamental in primary healthcare units, as the doctor has the right tools to triage the most serious cases. The results were promising as the ELF numerical value increased in line with the progression of PPF patterns.

## Introduction

Schistosomiasis is considered one of the main causes of fibrogenic liver disease and affects more than 250 million people worldwide [1]. Considered a neglected disease, it occurs predominantly in low- and middle-income countries. *Schistosoma mansoni* is the most relevant species in Brazil, which is the most affected country in the Americas [2].

In Pernambuco, an endemic state in Northeastern Brazil, according to data from the Schistosomiasis Surveillance and Control Program (SISPCE), in 2016, 9% of the 53 localities that carried out mass drug administration (MDA), presented sustained rates of hepatosplenic infection equal to or greater than 1% [3]. The World Health Organization (WHO), in line with the United Nations 2030 Agenda, established in 2015, has set a goal of eliminating schistosomiasis as a public health problem worldwide, with transmission reduced in at least 25 of the 78 endemic countries and the prevalence of hepatosplenic infection reduced to less than 1% during this period [4].

Diagnosing the presence and intensity of periportal fibrosis (PPF) is fundamental for preventing its complications, especially in patients with more advanced patterns of PPF, who could develop portal hypertension and are subject to upper gastrointestinal bleeding (UGIB) [5].

The PPF pattern is related to the host’s immune response and the parasite burden, causing the formation of granulomas in the portal spaces and PPF around them [6,7]. Ultrasonographic (US) assessment of PPF has become the technique of choice for measuring the PPF pattern in patients with *schistosomiasis mansoni* (SM) [8]. However, US is not available in primary healthcare units in endemic zones, and its use requires a trained and experienced examiner [9,10].

Ultrasonography allows comprehensive assessment of the liver parenchyma (PPF pattern), identifying portal hypertension, associated liver diseases and splenomegaly [11]. In fact, some studies have revealed an association between the measurement of PPF by ultrasonography and the serum levels of biomarkers, suggesting that the US findings reflect the pattern of PPF [9,11,12]. In 1996, a reproducible PPF classification was developed in Niamey, using PPF imaging standards ranging from patterns A to F [8]. The extent of PPF is related to the clinical form of the disease and correlates with portal pressure, such as esophageal varices occurring in patients with more advanced fibrosis or hepatosplenic form [7]. Another way of assessing liver fibrosis is through direct or indirect markers of fibrosis, used alone or together, known as indices [12]. These biological indices are more objective and have high reproducibility and numerical expression [13]. They have been widely used in recent years in viral hepatitis and fatty liver disease, which can progress to cirrhosis [12]. Commonly used indices include the Aminotransferase-to-Platelet Ratio Index (APRI), Fibrosis-4 Index (FIB-4), and Enhanced Liver Fibrosis (ELF), which show a good correlation with the histological assessment of fibrosis by METAVIR [14–17]. However, biological indicators can have some limitations, since they depend on the time of sample collection, vary widely, and are not specific.

However, studies evaluating the performance of biological fibrosis markers in measuring PPF in SM remain scarce [18]. Some studies have described isolated biomarkers, such as hyaluronic acid and platelet count, for assessing PPF, with Niamey’s classification by ultrasound as the reference standard [19]. A recent study reported a correlation between the degree of hepatic fibrosis of the APRI and FIB-4 in SM patients in a sample of 17 patients [20]. More recently, the Coutinho index, which incorporates serum alkaline phosphatase levels and platelet count, was proposed and demonstrated good accuracy in differentiating patients without PPF from those with advanced PPF [21].

The Enhanced Liver Fibrosis (ELF) index combines three markers in its calculation: hyaluronic acid, tissue inhibitory factor of metalloproteinases-1 (TIMP-1) and amino-terminal propeptide of type III procollagen (PIIINP). It assesses fibrosis through the metabolism of the hepatic cellular matrix in a direct, precise, automatic, and reproducible way [17]. This index has prognostic value in the early stages of hepatitis B and C or autoimmune hepatitis and can predict clinical outcomes better than biopsy [13]. In a systematic review including patients with alcoholic liver disease and metabolic dysfunction-associated steatotic liver disease (MASLD), ELF showed excellent performance in identifying advanced fibrosis, making it an alternative to liver biopsy in selected patients with chronic liver disease [22]. These findings in MASLD were corroborated by a meta-analysis [23], showing that ELF has high sensitivity and a high negative predictive value, especially in the primary care population, with the ability to exclude advanced fibrosis.

This study aims to evaluate the association between the ELF index and the patterns of periportal fibrosis established by ultrasound based on the Niamey classification and the history of UGIB in patients with SM, while also describing their sociodemographic and clinical profiles.

## Methods

### Ethics statement

This study was approved by the Human Research Ethics Committee of the Hospital das Clínicas de Pernambuco under protocol number CAAE: 57428422.8.0000.8807. Informed consent for the research was obtained through the signing of the written Informed Consent Form by each participant.

A cross-sectional, bidirectional analytical study was conducted between 2020–2023, including 196 patients infected with SM to evaluate the association between the values obtained using the ELF serological index and the PPF pattern determined by abdominal US according to the Niamey classification. Sociodemographic and clinical data were also evaluated.

Patients aged 18 years or older with a diagnosis of SM based on a clinical history of contact with water sources in endemic areas, a positive stool test for *S. mansoni* eggs or previous treatment with praziquantel, as well as upper abdominal US findings of PPF, were included. Patients with active or cured viral hepatitis B or C, steatotic liver disease, alcoholic or drug-induced liver disease, hepatocarcinoma, or splenectomized were excluded.

The sociodemographic (age and sex) and clinical data (history of upper gastrointestinal bleeding, US pattern of PPF database), as well as the biorepository containing biological samples from patients selected for the validation of the Coutinho index in a previous project, were evaluated [23].

In addition, patients with more advanced PPF (patterns D, E and F) were also evaluated. These patients were followed up at the Schistosomiasis outpatient clinic at the Hospital das Clínicas of the Universidade Federal de Pernambuco (HC-UFPE), from April to August 2022. All participants met the same inclusion and exclusion criteria for the previous project [21] and underwent abdominal US to confirm the diagnosis and exclude other liver diseases, as well as serum collection.

Sociodemographic and clinical data were collected, and an upper abdominal US was performed using a Siemens Acuson S2000 (Siemens Medical Solutions, Mountain View, CA, USA) with a 6C1 MHz transducer. US was performed by the same experienced operator in all included patients using the Niamey classification for categorizing PPF (A, no fibrosis; B, suspected fibrosis; C, mild; D, moderate; E, advanced; and F, very advanced) [24]. Following US, 10 mL of blood was collected from a peripheral vein, centrifuged, aliquoted and frozen at - 80º C in the laboratory of the Aggeu Magalhães Research Institute. The frozen serum samples were sent to the Central Laboratory of the Real Hospital Portugues for measurement of the ELF index [13].

For data analysis, the sample was divided into five groups according to Niamey’s classification: 18 with pattern A + B, 64 with pattern C, 56 with pattern D, 49 with pattern E, and 9 with pattern F. The variables involved were tested for normality and homogeneity using the Shapiro-Wilk and Bartlett tests, respectively.

The Student’s t-test was used when normality was observed in the data, to evaluate the mean differences, between the mean serum ELF value of the five groups. However, when the assumption of normality was violated, the Mann-Whitney test was used to analyze the medians. ANOVA test with post hoc (Tukey’s Honestly Significant Differences (HSD) test) was employed when the assumption of homogeneity was met, to assess the differences in means. However, when the assumption of homogeneity was violated, the Kruskal-Wallis test with post hoc (Fisher’s Least Significant Difference (LSD) test) was used to assess the difference in medians.

Pearson chi-square test was used for qualitative data, and Fisher’s exact test was used when necessary. The data were also evaluated using the ROC curve. A 5% significance level was set to confirm the conclusions. The R software (R DEVELOPMENT CORE TEAM, 2020) was employed to evaluate the results of the study.

## Results

The study included 196 patients, of whom 114 (58.2%) were female. The mean age was 45.04 years ± 15.30 years standard deviation (SD) and 20 (69%) individuals with advanced or very advanced PPF had a history of UGIB. The patients’ demographic and clinical data are described in Table 1. When comparing individuals with fibrosis according to age, statistically significant differences were observed (p < 0.0001), with differences noted between the mean values of C vs. D (p = 0.0126), C vs. E (p < 0.0001), and C vs. F (p = 0.0050) (Table 1). Regarding the history of UGIB, an increasing gradient of values was observed among patients with a history and a decreasing gradient for those without such a history, with statistical significance (p < 0.0001).

**Table 1:** Demographic and clinical characteristics of 196 patients with SM, distributed into five groups (Group A + B = no fibrosis; group C = mild fibrosis; group D = moderate fibrosis; group E = advanced fibrosis; group F = very advanced fibrosis) according to the pattern of PPF established by the Niamey ultrasound classification.

Characteristics	Group A + B	Group C	Group D	Group E	Group F	P-value
**N**	18 (9%)	64 (33%)	56 (28%)	49 (25%)	9 (5%)	
**Age (mean** ± sd)* Range	42.39 ± 13.21 (22 – 66)	38.02 ± 14.05 (18 – 66)	46.46 ± 14.27 (19 – 74)	51.57 ± 14.08 (24 – 79)	55.89 ± 19.26 (32 – 79)	< 0.0001**
**Sex** **Male (n, %)**	9 (50%)	24 (37%)	20 (36%)	23 (47%)	6 (67%)	0.3129***
**Female (n, %)**	9 (50%)	40 (63%)	36 (64%)	26 (53%)	3 (33%)	
**History of UGIB** **Yes**	0 (0%)	3 (5%)	6 (12%)	16 (48%)	4 (44%)	< 0.0001*
**No**	18 (100%)	61 (95%)	50 (88%)	33 (52%)	5 (56%)	

* Values in mean ± SD; UGIB: Upper gastrointestinal bleeding; ** Bartlett test - ANOVA test and post hoc Tukey HSD test; *** Fisher’s exact test.

Table 2 provides the serum ELF values. The mean ELF serum value increased according to the progression of PPF (p = 0.0021). Statistical differences were observed between the mean values of A + B vs. F (p = 0.0220), F vs. C (p = 0.0025), and F vs. D (p = 0.0295), no differences were observed among the other groups. There was no difference between patients with history of UGIB and the mean ELF serum value (p = 0.2274).

**Table 2:** ELF value of 196 patients with SM, distributed into five groups (Group A+ B  =  no fibrosis; group C  =  mild fibrosis; group D  =  moderate fibrosis; group E  =  advanced fibrosis; group F  =  very advanced fibrosis) according to the pattern of PPF established by ultrasound (Niamey classification) and history of upper gastrointestinal bleeding.

Groups of PPF^1^	Minimum value	Maximum value	Mean	SD	P-value
**A + B**	6.33	9.97	8.43	0.75	0.0021*
**C**	6.49	11.45	8.35	0.90	
**D**	6.53	11.42	8.62	0.96	
**E**	6.02	11.80	8.82	1.20	
**F**	8.31	11.52	9.67	0.96	
**History of UGIB** ^**2**^	**1st Q**	**3rd Q**	**Median**	**SD**	**P-value**
**No**	7.95	9.06	8.54	0,98	0.2274**
**Yes**	8.10	9.38	8.84	1,29	

Mean^1 (Min – Max)^ / Median^2 (1st Q - 3rd Q)^ / * Bartlett test - ANOVA test and Tukey HSD post hoc / ** Shapiro-Wilk test - Mann-Whitney test

Fig 1A shows the serum median (1st Quartile - 3rd Quartile) ELF value of 196 SM patients, distributed into four groups according to the pattern of PPF established by the Niamey classification, using US, with an increase according to the progression of PPF. The ELF index could differentiate between patients without PPF and those with mild, moderate and advanced PPF: A + B vs. C vs. D vs. E + F (p = 0.0024). ELF was able to differentiate patients without PPF from those with mild PPF (A + B vs. C) (p = 0.0002). It could also differentiate moderate PPF from advanced PPF (D vs. E + F) (p = 0.0411). Furthermore, ELF differentiated those without PPF from those with advanced PPF (A + B vs E + F) (p = 0.0454).

**Fig 1:**
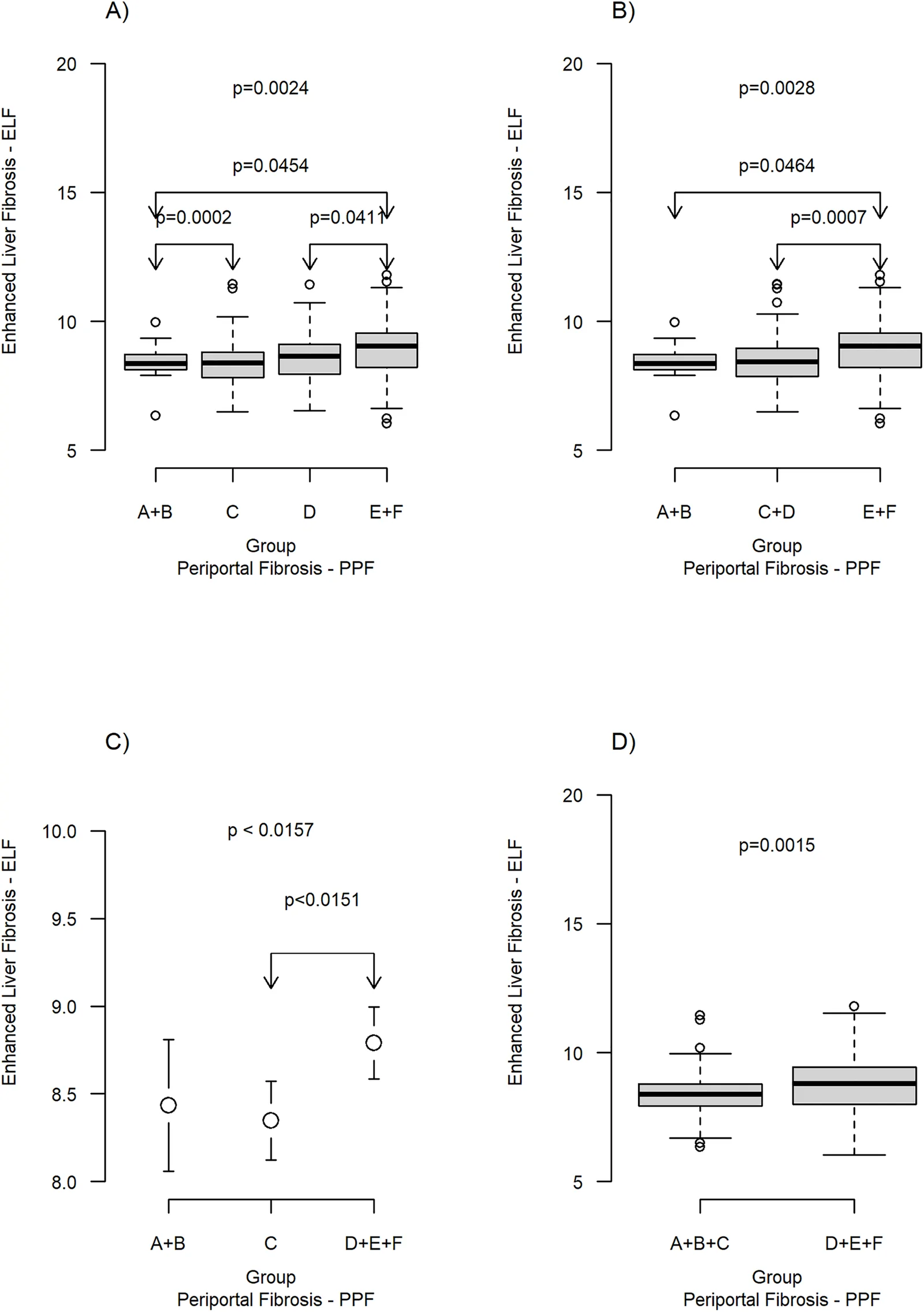
Serum ELF levels of 196 patients with schistosomiasis mansoni distributed into different groups. (A) (A + B vs C vs D vs E + F], (B) (A + B vs C + D vs E + F], (C) (A + B vs C vs D + E + F] and (D) (A + B + C vs D + E + F] according to the pattern of PPF, established by the Niamey classification, using ultrasound, Recife, 2023.

Fig 1B shows the serum median (1st Quartile - 3rd Quartile) ELF value of 196 SM patients, divided into three groups according to the PPF pattern established by the Niamey classification using US, with an increase according to the progression of PPF (p = 0.0028). The ELF index differentiated those without PPF from the mild or moderate and advanced patterns: A + B vs. C + D vs. E + F (p = 0.0028). ELF differentiated patients without fibrosis from those with advanced fibrosis: A + B vs. E + F (p = 0.0464), as well as those with moderate fibrosis from those with advanced PPF: C + D vs. E + F (p = 0.0007). The ELF index was unable to differentiate those without PPF from those with moderate (pattern A + B from pattern C + D) (p > 0.05).

Fig 1C shows the serum mean (CI 95%) ELF value of 196 SM patients, distributed into three groups according to the pattern of PPF established by the Niamey classification using US, separating the samples into groups without fibrosis, mild PPF, and moderate/advanced PPF: A + B vs. C vs. D + E + F. Once again, ELF was able to distinguish the different fibrosis groups with significance (p < 0.0157). There was also a differentiation between the mild fibrosis and moderate/advanced fibrosis groups: C vs. D + E + F (p < 0.0151).

Fig 1D shows the serum median (1st Quartile - 3rd Quartile) ELF value of 196 SM patients, distributed into two groups according to the pattern of PPF established by the Niamey classification, using US, separating the samples into groups with no or mild PPF and moderate or advanced PPF: A + B + C vs. D + E + F. The index was able to differentiate patients with absent or mild PPF from those with moderate or advanced PPF with statistical relevance: A + B + C vs. D + E + F (p = 0.0015).

(Fig 1A and 1B) Bartlett test – Kruskal-Wallis test with Fisher’s LSD test post hoc / (Fig 1C) Bartlett test – ANOVA test with post hoc Tukey HSD and (Fig 1D) Shapiro-Wilk test – Mann-Whitney test.

A ROC (Receiver Operating Characteristics) curve was constructed, and the AUROC (Area Under the Receiver Operating Characteristics) curve was calculated to measure diagnostic accuracy for identifying no or mild PPF (A + B + C) and moderate or advanced PPF (D + E + F), obtaining an AUROC = 0.633 (p = 0.0008) (95% CI; 0.561 – 0.700), with 53.98% sensitivity and 74.7% specificity, using a cut-off point of ELF > 8.75, as illustrated in Fig 2A. Fig 2A shows an AUROC curve representing the serum ELF value of 196 patients with schistosomiasis mansoni distributed into two groups (A + B + C vs D + E + F) according to the PPF pattern, established by the Niamey ultrasound classification, with ELF = 8.75 as the best cut-off point.

**Fig 2:**
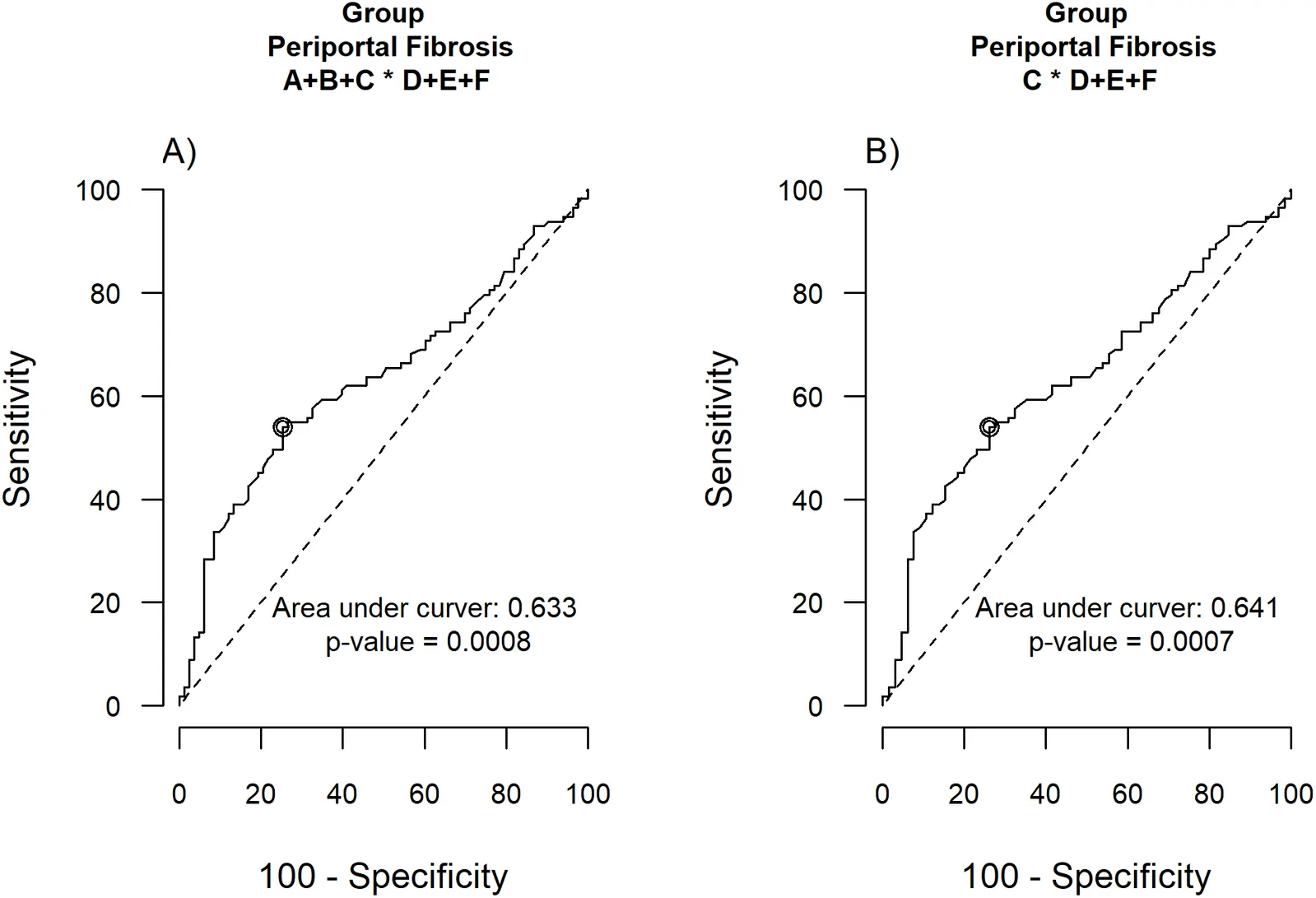
AUROC curve representing the serum ELF levels, distributed in groups according to the FPP pattern. (A) (A + B + C vs D + E + F) with ELF = 8.75 as the best cut-off point and (B) (C vs D + E + F) with ELF = 8.75 as the best cut-off point, Recife, 2023.

Fig 2B shows an AUROC curve representing the serum ELF value of 178 patients with schistosomiasis mansoni distributed into two groups (C vs. D + E + F) according to the PPF pattern, established by the Niamey ultrasound classification, with ELF = 8.75 as the best cut-off point.

## Discussion

As the regions affected by SM often have precarious infrastructure, this research aimed to evaluate strategies that can diagnose the pattern of PPF using easily applicable and reproducible biological tests. To date, there are no other published studies evaluating the performance of the ELF index in patients with SM. This study is the first to associate ELF values with PPF patterns according to the Niamey classification using US. In this study, ELF was able to differentiate patients without PPF, with mild, moderate and advanced PPF: A + B vs C vs D vs E + F, as well as patients with advanced PPF from those without fibrosis or with moderate fibrosis: A + B vs E + F and C + D vs E + F.

Additionally, this index could also differentiate patients with absent or mild PPF from those with moderate or advanced PPF: A + B + C vs D + E + F. In effect, studies have demonstrated that ELF is reproducible, feasible, and safe [22,23].

ELF was able to detect advanced fibrosis in patients with steatohepatitis associated with metabolic dysfunction in a study involving 3,207 patients of multiple ethnicities, with performance comparable to transient elastography and FIB-4 [25]. The good performance of ELF in the MASLD population was also described [26] in 829 patients, with similar performance in elderly patients (AUROC 0.74; 95% CI, 0.58 – 0.87).

Indeed, a systematic review [22] demonstrated the accuracy of ELF in diagnosing liver fibrosis in various etiologies of chronic liver disease, such as hepatitis B and C, MASLD, alcoholic liver disease and mixed etiologies, with excellent performance in MASLD (AUROC 0.78 – 0.97) and alcoholic liver disease (AUROC 0.92 – 0.94), as well as good performance in viral hepatitis (AUROC 0.69 – 0.98). However, in a study of 49 patients with chronic hepatitis C virus (HCV), despite predicting the degree of hepatic fibrosis, ELF performed less well than elastography [27]. Rasmussen et al [28] again evaluated ELF, comparing it with hepatic elastography, finding similar accuracy between the tests in a sample of 462 patients with compensated alcoholic liver disease. Thus, the authors recommended the use of ELF as a diagnostic tool in the early stages of alcoholic liver disease or compensated alcoholic cirrhosis.

In the present study, ROC curve analysis showed that using a cut-off point of ELF value > 8.75 in the groups of no PPF or mild PPF (A + B + C) *versus* moderate and advanced PPF (D + E + F), with 53.98% sensitivity and 74.7% specificity. While analyzing only patients with PPF, separating them into the mild and more advanced forms (C vs. D + E + F), with 53.98% sensitivity and 73.85% specificity, for the same ELF value > 8.75, it can be observed that both tests presented similar overall performance, with estimates of the probability of correct classification of a random subject with moderate accuracy, represented by AUROC = 0.633 and AUROC = 0.641, respectively, reflecting a chance of correct classification of around 63% of the time.

In a study involving 152 patients with chronic liver disease caused by chronic hepatitis B virus (HBV), ELF proved to be a non-invasive marker, replacing the indication for liver biopsy. The study used the manufacturer’s cut-off points to define the degree of fibrosis, characterizing fibrosis as mild (ELF < 7.7), moderate (ELF between 7.7 and 9.8) and advanced (ELF ≥ 9.8) [29]. Additionally, in the meta-analysis carried out [25], the ELF cut-off points were studied in individuals with MASLD, including 2,265 patients with advanced fibrosis (≥ F3). Using a cut-off value of 7.7, ELF showed high sensitivity (93%; 95% CI, 0.82 – 0.98), while raising the cut-off point to 9.8 showed high specificity (86%; 95% CI, 0.77 – 0.92) [23].

These ELF values, especially in relation to specificity, are compatible with those found in the assessment of PPF in our study, with a median finding of 9.75 in very advanced PPF (pattern F). Nevertheless, even in patients without PPF (A + B), the median ELF value was 8.36, above the value that would correlate with mild fibrosis in both MASLD and chronic HBV liver disease [23,29]. Our data on PPF in SM were partly similar to those described in patients with HCV liver disease [27], with ELF 8.85 for diagnosing significant fibrosis (F ≥ 2] (89.5% sensitivity) and 9.19 for advanced fibrosis/cirrhosis (F ≥ 3) (87.5% sensitivity).

In the context of portal hypertension, a study evaluated ELF in a sample of 201 patients with chronic liver disease composed mainly by alcoholic liver disease and viral hepatitis [30]. An ELF value ≥ 11.1 identified patients at risk of clinically significant portal hypertension, while ELF < 10.1 ruled out high risk portal hypertension ≥ 20 mmHg.

The progression of PPF leads to portal hypertension and its complications, such as the gastro-esophageal varices and UGIB, reported to occur in up to 80% of patients. UGIB is the main cause of mortality associated with schistosomiasis, with a mortality rate of up to 30% [31]. In the present sample, a higher frequency of UGIB was reported in patients with more advanced patterns of PPF, suggesting that these patients have higher serum ELF levels. However, there was no difference in the mean ELF serum value between patients with and without the history of UGIB. This finding may have been due to the small number of patients with a history of UGIB, one of the limitations of this study.

Additionally, the study population consisted of relatively younger patients (mean age was 45.04 years ± 15.30), in whom individuals with advanced or very advanced PPF had a history of UGIB. Nevertheless, not all patients with advanced fibrosis will develop esophageal varices, and not all patients with esophageal varices will have UGIB. In Brazil, for example, the frequency of UGIB is lower than that in Africa [5]. However, it is known that the sub-Saharan Africa accounts for 91% of all schistosomiasis cases, where predominately rural communities reside under impoverished conditions [7].

When evaluating non-invasive markers for assessing PPF, Santos JC et al [18] highlighted the limitation of APRI as being susceptible to interference from liver comorbidities, while the Coutinho index would require further studies, especially in populations with mild and moderate PPF [14,21].

A study involving 187 patients with schistosomiasis demonstrated an inverse correlation between the PPF pattern according to the Niamey classification and the platelet count (p < 0.001) (19). However, that study (19) did not evaluate the PPF patterns between patients with moderate (C + D) and advanced (E + F) patterns, as we did in the present article. In addition, the Coutinho index was identified as one of the most applicable non-invasive tests for assessing PPF in schistosomiasis mansoni, suggesting that they should be used as screening tools to identify patients who would require a more precise assessment using US or elastography [18].

The present study demonstrated that ELF index was able to identify the most advanced degrees of fibrosis, making ELF relevant to identify patients at risk of complications, and it could be used for this purpose. Therefore, in medical practice in endemic areas for SM, an ELF value above 8.75 would indicate more advanced patterns of PPF (D, E or F) and portal hypertension. These patients, who may have gastroesophageal varices and history of UGIB should be sent to referral centers, where they will be better assessed and appropriately treated.

The limitations of this study are primarily related to the small number of patients without PPF (pattern A + B) or with very advanced PPF (pattern F), which may have interfered with the statistical analysis. Although the distribution of patients followed a normal distribution (Gaussian), with fewer cases at the ends of the curve, the ELF was able to separate patients without PPF or with doubtful PPF (pattern A + B) from the other patterns, as well as separating those with advanced or very advanced PPF (pattern E + F) from the others with less PPF. This capacity becomes fundamental in primary healthcare units, as the doctor has the right tools to triage the most serious cases. The results were promising as the ELF numerical value increased in line with the progression of PPF patterns.

## Conclusion

The ELF index has demonstrated significant potential as a tool for assessing PPF in patients with SM, differentiating those with little fibrosis from those with moderate or advanced fibrosis. Further studies should be carried out correlating ELF with other biomarkers of liver fibrosis in SM, using PPF pattern established by US as a reference.
