# Supplementary material for: Evaluating periportal fibrosis in schistosomiasis mansoni using the ELF [Enhanced Liver Fibrosis] score: A pilot study
Source: PLoS Negl Trop Dis. 2026 Oct 9;20(10):e0014775. doi: 10.1371/journal.pntd.0014775 (PMC13671643; doi:10.1371/journal.pntd.0014775)
Supplement: Supplementary file 1 [file pntd.0014775.s002.doc]

**Ref: Major revision requested:** **PNTD-D-25-01595 - [EMID: f121d8073e60d153]**

Recife, February 18^th^, 2026.

**Dr. David J. Diemert, M.D**

**Academic Editor**

**PLOS Neglected Tropical Diseases**

Dear Dr. David J. Diemert, M.D,

We would like to express our sincere gratitude for the thoughtful comments and constructive suggestions provided regarding our manuscript. The feedback was instrumental in refining our work, improving its clarity, and strengthening the presentation of our results.

We are sending a revised version of the manuscript “Evaluating periportal fibrosis in schistosomiasis mansoni using the ELF [Enhanced Liver Fibrosis] score: a pilot study” by, Rebecca Dantas Thorp (ORCID 0000-0001-9628-3233), Andrea Dória Batista (ORCID 0000-0002-7996-5053), Carolline A Mariz (ORCID 0000-0002-7651-9429), Lilian Rose Maia Gomes de Araújo (ORCID 0000-0002-9643-5417), George Tadeu Nunes Diniz (ORCID 0000-0002-9620-262), Ana Virgínia Matos Sá Barreto (ORCID 0000-0003-3901-714), Clarice Neueschwander Lins de Morais (ORCID 0000-0002-0887-816), Paula Carolina Valença Silva (ORCID 0000-0002-9478-0743), Ana Lúcia Coutinho Domingues (ORCID 0000-0002-1824-7090) and Edmundo Pessoa Lopes (ORCID 0000-0002-3470-1564).

To facilitate the review process, we have provided two files: A) "Tracked Changes" version: Showing all modifications made to the original text. B) "Clean" version: With all changes accepted and formatted.

Additionally, we have updated the author contribution section following the CRediT (Contributor Roles Taxonomy), as requested in the journal guidelines. Furthermore, the list of Supplementary Information captions has been removed.

Below, we address the Reviewer's concerns point-by-point. Our responses are presented in Times New Roman font and blue color.

**Reviewer 1**

This paper presents a small study in a Brasilian schistosomiasis clinic where 196 patients were studied, and a new biomarker was measured in attempts of associating with image-based patterns. The study lacks comparison to other biomarkers, inadequate information to follow the study design (sampling justification, controlling for co-infections, justification for image pattern groupings, or quality control for image diagnoses). The discussion would require major rewriting to focus only on comparisons that are relevant to the data analyzed in this study. I suggest the title be rewritten to indicate this is a pilot study of ELF measurements in schistosomiasis patients.

**Author’s response:**

We sincerely thank the reviewer for the constructive criticisms, which provided an opportunity to significantly enhance the methodological rigor and clarity of our manuscript. In response to the concerns raised, we have thoroughly revised the study's title to explicitly state its nature as a pilot study: 'Evaluating periportal fibrosis in schistosomiasis mansoni using the ELF (Enhanced Liver Fibrosis) score: a pilot study'.

Regarding the study design, we have expanded the Methods section to clarify that we utilized a sample of 196 patients, which is appropriate for the exploratory objectives of this pilot investigation. To ensure the specificity of our findings and address the issue of co-infections, we have now explicitly detailed our strict exclusion criteria, which ruled out patients with viral hepatitis (B and C), steatotic liver disease, and alcoholic or drug-induced liver injury. Quality control for image diagnoses was addressed by clarifying that all ultrasonographic assessments were performed by a single senior sonographer, following the Niamey classification, before ELF results.

Finally, the Discussion section has been rewritten to focus on comparisons relevant to our data, including a comparative analysis with other non-invasive biomarkers, as reported in the current literature for schistosomiasis.

Introduction

- Line 70: What is meant by serious infections? Define. Again, what is a severe infection? Line 74. Use exact terminology of infection intensities.

**Author’s response:**

Thanks for this suggestion. Severe infection was defined as the “hepatosplenic” infection, as you can see in yellow changes in the Introduction section.

- The introduction should be rewritten to focus on the different indicators of fibrosis and a direct comparison, including citing studies that have shown their past agreement. A recent review, in their data extraction table, note all studies performing both biomarker analyses and imaging and their agreement. <https://journals.plos.org/plosntds/article?id=10.1371/journal.pntd.0012033>

**Author’s response:**

We sincerely appreciate the reviewer’s recommendation to include the recent systematic review published in PLOS Neglected Tropical Diseases [<https://journals.plos.org/plosntds/article?id=10.1371/journal.pntd.0012033>]. This high-quality evidence was instrumental in restructuring our Introduction to provide a more critical comparison between imaging and biochemical indicators. As suggested, we have modified the Introduction to emphasize the current landscape of fibrosis assessment in schistosomiasis, specifically highlighting the agreement levels and limitations of existing biomarkers.

- Biological indicators are not necessarily better, given they are reliant on the time point of the sample, vary widely, and are non-specific. Please indicate these limitations. Also, give examples of associations (strength, direction, population studied) of the indices quoted from past studies (Lines 95-96).

**Author’s response:**

We appreciate the reviewer’s grounded perspective on the limitations of biomarkers. We have revised the Introduction to explicitly acknowledge that biological indicators may be influenced by the timing of sample collection and lack disease specificity.

- Line 100: Issues in acknowledging a gold/reference standard for fibrosis are needed to be acknowledged. Niamey is by no means a gold standard.

**Author’s response:**

We thank the reviewer for this critical observation regarding the terminology of 'gold standard'. We agree that while the Niamey protocol is the most widely utilized tool in schistosomiasis research, it carries significant limitations, including operator dependence and challenges in reproducibility, as highlighted by recent paper [Ockenden et al., 2024]. Moreover, the study by King et al., 2003, reported that measurements of the walls of the segmented portal branches were time-consuming and poorly reproducible. Consequently, we have replaced the term 'gold standard' with 'reference standard' throughout the manuscript to avoid overestimating the accuracy of ultrasonography.

**Methods**

- How were patients sampled? What was the selection strategy? Provide information on the sampling register choice, strategy, and power calculations.

**Author’s response:**

Thank you for the observation. In fact, patients with PPF patterns A, B, and C were recruited from endemic areas in our region, while individuals with advanced patterns (PPF D, E, and F) were recruited at the Schistosomiasis Outpatient Clinic of the Hospital das Clínicas (UFPE). All met the previously established inclusion and exclusion criteria and underwent abdominal US for diagnostic confirmation, exclusion of other liver diseases, and obtaining serum samples, as described in reference 21.

Sample size calculation was performed to evaluate the sensitivity and specificity of the indices, using the formula N = Z² [P (1 − P)] / D² for validation studies (Andrade; Zicker, 1997), considering P = 95%, D = 0.05, and Z = 1.96. The estimated sample was 292 patients, equally distributed into four groups (A+B, C, D, E+F). However, limitations in serum samples and difficulties in finding patients from extreme groups (pattern A+B and pattern E+F) resulted in a final sample of 196 patients.

-Give the frequency range of the transducer.

**Author’s response:**

Upper abdominal US was performed using a Siemens Acuson S2000 (Siemens Medical Solutions, Mountain View, CA, USA) with a 6C1 MHz transducer. It was always performed by the same experienced operator (ALC Domingues), coauthor of this study.

- Was ultrasound performed by a sonographer who knew the ELF results? If so, this setup is a limitation and could have biased the sonographer’s image results. It should be acknowledged in the discussion.

**Author’s response:**

Upper abdominal ultrasound was performed by an experienced operator who was unaware of the ELF results. Indeed, ELF measurement was performed after US scan.

In the Methods section (line 188), it was described: “Following US, 10 mL of blood was collected from a peripheral vein, centrifuged and aliquoted...to be measured for the ELF index [13].”

- Was any re-reading of the ultrasound images done to assess the quality of the diagnosis? If not, acknowledge as a limitation.

**Author’s response:**

Great questioning. In this study, no re-reading of the Niamey classification results evaluated by ultrasonography was performed for the following reasons. All US scan were performed by the same operator (ALC Domingues), who has extensive experience of more than 30 years, with some studies published on ultrasonography in schistosomiasis mansoni, using the Cairo and later Niamey classifications. Dr. Domingues participated in the Second satellite symposium on ultrasound in schistosomisis in Belo Horizonte, Brazil (*Richter J, Domingues ALC, Barata CH, Prata AR, Lambertucci JR. Report on the second satellite symposium on ultrasound in schistosomiasis. Mem Inst Oswaldo Cruz. 2001; 96:151-6*). Due to all this expertise, we suppose that our Niamey classification results presented satisfactory accuracy, and we do not consider that the lack of re-reading of the US would be considered a limitation of this study.

- Were any of the distributions of ELF normal? If not, just describe the statistical test actually used.

**Author’s response:**

Great questioning. In this study. Now, all the tests used are reported below (tables/figures), as you can see in yellow color in Results section.

- Do you mean chi2 was used for categorical data? When was fisher’s exact p-value calculated? Be specific.

**Author’s response:**

Table 1 (Sex x PPF and History of UGB x PPF categorical data) were performed Fisher´s exact test, in yellow.

-Indicate R version.

**Author’s response:**

Lines 206-207 indicated R version and the complete citation it below

R Core Team (2020). _R: A Language and Environment for Statistical Computing_. R Foundation for Statistical Computing, Vienna, Austria. <https://www.R-project.org/>.

-Why wasn’t ELF compared to other biomarkers or indices?

**Author’s response:**

Thanks for this observation. However, we did not find robust studies in the literature describing the PPF induced by *Schistosoma mansoni* measurement through serum biomarkers. In addition, some articles involve other species of Schistosoma or the schistosomiasis coinfcetion with viral hepatitis.

Yet, in the Introduction section we describe two studies, one involving the measurement of PPF by APRI and FIB-4 with few schistosomiasis patients (lines 137 to 139). The other study describes the validation of the Coutinho index, which was created by our group, both in the Introduction and in the Discussion sections (reference 21).

In addition, we add into the Discussion section a paper describing the inverse relationship between the Niamey PPF pattern and platelet count, reference 19, (lines 367 to 370).

- ELF needs to be better introduced, explaining it’s function and biological measurement.

**Author’s response:**

Sorry, but we do not understand this suggestion. ELF was introduced and its operation explained in the penultimate paragraph of the Introduction section, from line 142 to 153. We also cite some references from its creation to its clinical application in robust studies: 13, 17, 22 and 23 in the manuscript.

**Results**

- What is the p-value here comparing?! Lines 170-173. Give values of both comparison groups. It doesn’t make sense to put p-values on simple descriptives of prevalence unless something is being compared. Please write out the comparison groups.

**Author’s response:**

Thank you for the observation. In fact, it was corrected in the text, leaving only the p-values where it actually makes sense, as you can see in yellow color in Results section.

- Table 2: Show the standard deviations not the min and max values. We need to know what is being compared. Also, why does it say mean in the column header and median in the footnote? What is being compared?? If median then show the median and the interquartile range. If mean then show mean and standard deviation. It is not clear the right statistical tests were used. We also need to see the observations/patients in each category compared.

**Author’s response:**

Thank you for the observation. The text was corrected to provide greater clarity in the description of the result, and the tests used were also listed. as you can see in yellow color in Results section.

- Fig. 1 why are there no differences between a+b vs c? Why is a+b higher than c?
- A, which represents normal should be analyzed separately. This would allow answering the question as to whether ELF differentiates between normal and fibrosed. Then the next question is whether it differentiates patterns associated to periportal fibrosis.

**Author’s response:**

Fig 1A

|  | **N** | **Min** | **Max** | **Mean** | **Median** | **SD** | **p1-value** | **P2-value** | **Sig** | **P3-value** |
| --- | --- | --- | --- | --- | --- | --- | --- | --- | --- | --- |
| **ELF** |  |  |  |  |  |  |  |  |  |  |
| A+B | 18 | 6 | 10 | 8.43 | 8.36 | 0.75 | 0.0500 | **0.0024** | A+B - E+F | **0.0454** |
| C | 65 | 6 | 11 | 8.35 | 8.40 | 0.90 |  |  | C - E+F | **0.0002** |
| D | 55 | 7 | 11 | 8.62 | 8.64 | 0.96 |  |  | D - E+F | **0.0411** |
| E+F | 58 | 6 | 12 | 8.95 | 9.04 | 1.20 |  |  |  |  |

A+B x C The values are so close (8.36 x 8.40) and test (Kruskal-Wallis post hoc Fisher’s Least Signficant Diference (LSD) test has no found difference statistics.

|  | **N** | **Min** | **Max** | **Mean** | **Median** | **SD** | **p1-value** | **p2-value** | **Sig** | **p3-value** |
| --- | --- | --- | --- | --- | --- | --- | --- | --- | --- | --- |
| **ELF** |  |  |  |  |  |  |  |  |  |  |
| A+B | 18 | 6.33 | 9.97 | 8.43 | 8.36 | 0.75 | 0.0646 | **0.0157** | C x D+E+F | **0.0152** |
| C | 65 | 6.49 | 1.14 | 8.35 | 8.40 | 0.90 |  |  |  |  |
| D+E+F | 113 | 6.02 | 11.80 | 8.79 | 8.80 | 1.10 |  |  |  |  |

**p1-value - Bartlett test / p2-value - ANOVA test / p3-value – post hoc Tukey test**

When evaluating the averages, it can be observed that A+B is slightly above C. However, no statistical significance was observed between (A+B x C).

-Fig 2. Where are the regression models for these results??

**Author’s response:**

Thanks for this question. To evaluate the ROC curve, the software performs logistic regression in the background. However, it does not provide the regression model parameters themselves. Instead, it reports the area under the curve (AUC), standard error, 95% confidence interval, negative likelihood ratio, positive likelihood ratio, sensitivity, specificity, and the p-value.

**Discussion**

- Line 256: Why are you discussing fatty livers here and you haven’t presented any data in this are?

**Author’s response:**

Thank you for this question. We present data on ELF performance in patients with MASLD because these are the ones that have been published in the most robust studies, involving hundreds of patients (references 25 and 26 in the manuscript). We did not describe studies with ELF in schistosomiasis patients for PPF measurement because we did not find them in the literature. To our knowledge, this is the first study conducted to measure PPF in schistosomiasis patients.

- What is being claimed that ELF is associated with? Many disconnected examples are provided, but it does not appear that this study actually looked at co-morbidities or co-infections, so we have no real idea if it is distinguishing schistosomal liver fibrosis.

**Author’s response:**

Excellent question, thanks. All of these 196 patients, who were included in this study as schistosomiasis patients, did not report alcohol consumption or hepatotoxic drugs, had negative viral and autoimmunity markers. Moreover, in the US scan they had PPF, left liver lobe enlargement, and there were no indications of fat liver disease or cirrhosis. These inclusion/exclusion criteria are described in the Methods section. Therefore, in this study, serum ELF levels certainly were associated with PPF due to *S mansoni*.

- It is a limitation that this study has not been compared to other biomarkers.

**Author’s response:**

Thanks for this observation. However, we did not find robust studies in the literature describing the PPF evaluation by *S mansoni* through serum biomarkers. Some articles involve other species of Schistosoma or the schistosomiasis coinfection with viral hepatitis.

Yet, in the Introduction section we describe two studies, one involving the measurement of PPF by APRI and FIB-4 in a few schistosomiasis patients (lines 137 to 139). The other study describes the validation of the Coutinho index, which was created by our group, both in the Introduction and in the Discussion section (reference 21).

In addition, we add into the Discussion section a paper describing the inverse relationship between the Niamey PPF pattern and platelet count, reference 19, (lines 367 to 370).

Minor

- The manuscript needs to be read for grammatical errors, typos, and fluency in language.
**Author’s response:**

Thanks very much for this suggestion. We proofread the entire manuscript and corrected grammatical and typo errors, as well as improved fluency, in yellow.

-Avoid paragraphs of fewer than three sentences.

**Author’s response:**

Thanks for this suggestion. The manuscript has been revised, and all paragraphs in the text have at least three sentences.

-Spell out all abbreviations on first usage.

**Author’s response:**

Thanks for this suggestion. The manuscript has been revised, and all abbreviations were described the first time they are used.

-Shorten Table, etc titles.

**Author’s response:**

Thanks for this suggestion. Table titles have been revised and abbreviated.

- Upper GI bleeding = UGIB not UGB.

Author’s response:

We appreciate this correction. We have standardized the abbreviation to UGIB throughout the entire manuscript, including all tables, figures, and the main text.

Rebecca Dantas Thorp

Corresponding author
