# Supplementary material for: Evaluating periportal fibrosis in schistosomiasis mansoni using the ELF [Enhanced Liver Fibrosis] score: A pilot study
Source: PLoS Negl Trop Dis. 2026 Oct 9;20(10):e0014775. doi: 10.1371/journal.pntd.0014775 (PMC13671643; doi:10.1371/journal.pntd.0014775)
Supplement: Supplementary file 2 [file pntd.0014775.s003.doc]

**Ref: Major revision requested:** PNTD-D-25-01595R1**- [EMID: e6ffbc26126d54ee]**

Recife, July 5, 2026

**Dr. David J. Diemert, M.D**

**Academic Editor**

**PLOS Neglected Tropical Diseases**

Dear Dr. David J. Diemert, M.D,

We would like to express our sincere gratitude for the thoughtful comments and constructive suggestions regarding our manuscript. The feedback was instrumental in refining our work, improving its clarity, and strengthening the presentation of our results.

We are sending a revised version of the manuscript (PNTD-D-25-01595R1) “Evaluating periportal fibrosis in schistosomiasis mansoni using the ELF [Enhanced Liver Fibrosis] score: a pilot study”.

To facilitate the review process, we have provided two files: A) "Tracked Changes" version: Showing all modifications made (yellow) to the original text. B) "Clean" version: With all changes accepted and formatted.

Additionally, we have updated the author contribution section following the CRediT (Contributor Roles Taxonomy), as requested in the journal guidelines. Furthermore, the list of Supplementary Information captions has been removed.

Below, we address the Reviewer's concerns point-by-point. Our responses are presented in Times New Roman font and blue color.

**Reviewer 1**

The reviewers have raised several important concerns regarding this study that require a comprehensive response from the authors. In particular, one reviewer identified potential overlap between the present manuscript and previously reported work, raising questions about the novelty of the study, the relationship between the datasets, and the authorship of related publications. Please provide a detailed point-by-point clarification addressing all issues raised by the reviewers. Special attention should be given to the following comment:

Why was this 2017 study not referenced and compared: Diagnosing schistosomiasis-induced liver morbidity: implications for global control? The Ozaki et al. (2024) study is presented only as a conference abstract (A-250 Evaluation of Liver Fibrosis in Schistosomiasis Mansoni Using the ELF [Enhanced Liver Fibrosis] Score). Why is Ozaki not listed as an author on the present publication or on the related abstract available at: <https://www.apm.org.br/wp-content/uploads/SPMJ_v142Suppl1.pdf>?

Given the differences in authorship across these reports, it is unclear who was involved in the study and how the various datasets and publications are related.”

The authors should clearly explain the relationship between the current manuscript and any previously published articles, abstracts, conference presentations, or related reports, including whether the same study population, dataset, or analyses were used. In addition, the authors should clarify the contributions of all individuals involved and address any apparent discrepancies in authorship across related publications.

Author’s response:

We thank the reviewer for the comment.

We would like to clarify that Dr Bianca Ozaki contributed to the implementation and availability of the Siemens ELF test.

So, after the completion of the laboratory testing and data analysis, the abstract was sent to Dr Ozaki. She subsequently asked whether it could be presented at the 56^th^ Brazilian Congress of Clinical Pathology/Laboratory Medicine, held in Salvador, Bahia, Brazil. As none of the authors of this manuscript were able to present the abstract at the congress in Salvador, we approved its presentation by Dr Ozaki, which required the inclusion of her name as a co-author of the Congress abstract.

However, Dr Ozaki did not participate in the laboratory testing, data analysis, interpretation of the results, or preparation of the manuscript. Therefore, her name has not been included among the authors of the full-length paper submitted for publication.

Moreover, we did not cite the abstract presented in Salvador because we believe that conference abstracts should not be included in the reference list of a paper.

1)We note that your Data Availability Statement is currently as follows: "We confirm that all data reported in the manuscript are publicly available. The authors are making all reported data and metadata related to the study results available, as provided in the submission. All data can be found in the results section of the manuscript, as well as in the Supporting Information section.". Please confirm at this time whether or not your submission contains all raw data required to replicate the results of your study. Authors must share the “minimal data set” for their submission. PLOS defines the minimal data set to consist of the data required to replicate all study findings reported in the article, as well as related metadata and methods ([https://journals.plos.org/plosone/s/data-availability#loc-minimal-data-set-definition](https://journals.plos.org/plosone/s/data-availability#loc-minimal-data-set-definition)).

For example, authors should submit the following data:

1) The values behind the means, standard deviations and other measures reported;

2) The values used to build graphs;

3) The points extracted from images for analysis.

Author’s response:

We thank the reviewer for this valuable comment. The requested revisions have been implemented in the revised manuscript. Specifically, the underlying values for the reported means, standard deviations, and other summary measures, the data used to generate the graphs, and the data points extracted from images for analysis have been included. These modifications are highlighted in yellow throughout the manuscript.

**Reviewer 2:**

This is a straight forward but interesting study. I was not involved with the first review. Some lack of literature analysis appears to be the case. Why was this 2017 study not reference and compared “Diagnosing schistosomiasis-induced liver morbidity: implications for global control”

The ozaki paper 2024 study is a conference abstract only “A-250 Evaluation of Liver Fibrosis in Schistosomiasis Mansoni Using the ELF (Enhanced Liver Fibrosis) Score”, why is Ozaki not on this publication Or here too?

<https://www.apm.org.br/wp-content/uploads/SPMJ_v142Suppl1.pdf>
Again with different authors, so it is not clear who is actually involved with this study.

Author’s response

We thank the reviewer for the comment.

We would like to clarify that Dr Bianca Ozaki contributed to the implementation and availability of the Siemens ELF test.

So, after the completion of the laboratory testing and data analysis, the abstract was sent to Dr Ozaki. She subsequently asked whether it could be presented at the 56^th^ Brazilian Congress of Clinical Pathology/Laboratory Medicine, held in Salvador, Bahia, Brazil. As none of the authors of this manuscript were able to present the abstract at the congress in Salvador, we approved its presentation by Dr Ozaki, which required the inclusion of her name as a co-author of the Congress abstract.

However, Dr Ozaki did not participate in the laboratory testing, data analysis, interpretation of the results, or preparation of the manuscript. Therefore, her name has not been included among the authors of the full-length paper submitted for publication.

Moreover, we did not cite the abstract presented in Salvador because we believe that conference abstracts should not be included in the reference list of a paper.

**Reviewer 3**

The ultrasound PPS scoring system (A-F) needs to be better explained in the Methods. In the Results section, it becomes very confusing when the authors alternately refer to "no PPS", "mild or moderate PPS", vs. the letter designations. For this reviewer who is not an expert on ultrasound or portal hypertension, it took several reads to understand that grades A+B are equivalent to "no PPS" (I think). The severity definitions of each letter grade need to be spelled out.

Author’s response

We sincerely thank the reviewer for the constructive comments, which provided an opportunity to improve the methodological rigor and clarity of our manuscript.

In response to the concerns raised, we reiterate that the severity of each pattern is specified according to the Niamey classification system for PPF categorization [24]. As stated on line 185, ultrasound examinations were consistently performed by the same experienced operator in all included patients, using the Niamey classification for PPF categorization (A, no fibrosis; B, suspected fibrosis; C, mild; D, moderate; E, advanced; and F, very advanced) [24].

- Lines 168-169: it appears that the participants were classified as having had Sm based only on history of prior contact with water or treatment with praziquantel, without any confirmation of diagnosis with a parasitologic test (e.g., stool microscopy and/or serology). This likely resulted in inclusion of individuals who did not in fact have Sm. The authors should comment on this limitation for bias being introduced to the results based on the definition of Sm infection.

Author’s response

We thank the reviewer for this suggestion. Participants were classified as having schistosomiasis mansoni (SM) based on a history of prior contact with water sources, a positive stool test for *S. mansoni*, or treatment with praziquantel. As described in the Methods section (line 168): “Patients aged 18 years or older with a diagnosis of SM based on a clinical history of contact with water sources in endemic areas, a positive stool test for *S. mansoni* eggs or previous treatment with praziquantel, as well as upper abdominal US findings of PPF, were included.”

In addition, most of these patients included in the study have had an infection for some years, which evolved with periportal fibrosis and portal hypertension. During the initial diagnosis of *S. mansoni* infection, in the endemic area, they may have presented eggs in the fecal examination, were treated with praziquantel and instructed to avoid re-exposure to the worm. Following these years of follow-up, therefore, the females were eliminated by treatment or stopped oviposition with aging.

It is unclear why Table 2 needs 4 panels since it's looking at the same data just grouped together into different bins. Nothing more is added by panels C and D that isn't already evident from panels A and B.

- At no point in the Results is it pointed out that the ELF values in the Niamey C group were actually lower than A+B. It is a bit misleading to state that these two groups were significantly different when in fact the difference goes against the main finding that increasing ELF scores correlate with increasing PPS on ultrasound. The discrepancy between A+B and C needs to be clarified.

Author’s response

ANOVA was performed (p = 0.0021), followed by Tukey's post hoc test, comparing the means between patterns A + B vs C (p = 0.9976). This information has been added to lines 225–227: “Statistical differences were observed between the mean values of A+B vs. F (p=0.0220), F vs. C (p=0.0022), and F vs. D (p= 0.0295), no differences were observed among the other groups”.

The detailed results of Tukey's post hoc test are provided below:

> ELF.aov <- aov(ELF ~ factor(GrupoFPP1), db ); summary(ELF.aov) ; TukeyHSD(ELF.aov)

Df Sum Sq Mean Sq F value Pr(>F)

factor(GrupoFPP1)	4	17.3	4.326	4.384 0.00205 **

Residuals	191 188.5	0.987

---

Signif. codes: 0 ‘***’ 0.001 ‘**’ 0.01 ‘*’ 0.05 ‘.’ 0.1 ‘ ’ 1

1 observation deleted due to missingness

Tukey multiple comparisons of means

95% family-wise confidence level

Fit: aov(formula = ELF ~ factor(GrupoFPP1), data = db) $`factor(GrupoFPP1)`

diff	lwr	upr	p adj

C-A+B -0.08555569 -0.81419576 0.6430844 p = 0.9976178

D-A+B 0.18359601 -0.55927048 0.9264625 p = 0.9604355

E-A+B 0.38795292 -0.36604482 1.1419507 p = 0.6174779

F-A+B 1.23513238 0.11829148 2.3519733 **p = 0.0220128**

D-C	0.26915170 -0.23205808 0.7703615 p = 0.5774308

E-C	0.47350861 -0.04405608 0.9910733 p = 0.0905534

F-C	1.32068808 0.34770595 2.2936702 **p = 0.0022550**

E-D	0.20435691 -0.33305120 0.7417650 p = 0.8329291

F-D	1.05153638 0.06785529 2.0352175 **p = 0.0295962**

F-E	0.84717946 -0.14493467 1.8392936 p = 0.1334362

- Why do the authors present mean ELF scores in Table 1 but use medians for Figure 2? It makes no sense to use different measures.

Author’s response

We sincerely thank the reviewer for the comment, which contributed to improving the manuscript. In Table 1, Bartlett's test was used to assess the homogeneity of variances among the groups and to define the most appropriate statistical test to verify the presence or absence of differences between means or medians. For the analysis presented in Figure 1, homogeneity of variances and normality of the data were assessed in order to determine whether a parametric or non-parametric approach should be used. These procedures are described in the Methods section (lines 193 to 203). The following references were used: CONOVER W.J. **Practical Nonparametrics Statistics**., 1980 and ZAR J. H. **Biostatistical Analysis**., 2nd. Ed. 1984

In Figure 2, means and medians were not used. This figure presents the ROC curve, based on logistic regression, which provides the odds ratio (OR) as a measure of association, expressing the probability of occurrence or non-occurrence of the event under study. The following references were used: HOSMER D. W. **Applied Logistic Regression**., 2nd ed, 2000 and ZHOU X.H. **Statistical Methods in Diagnostic Medicine**., 2002.

- Lines 213-215: it would be helpful not just to say that there was a statistically significant difference in age between the PPF levels, but that increasing PPF score was associated with increasing age (in general).

Author’s response

We appreciate this valuable suggestion. We have revised the manuscript to include the main differences in mean age among the PPF pattern groups. The following sentence has been added: "When comparing individuals with fibrosis according to age, statistically significant differences were observed (p < 0.0001), with significant differences between the mean values of C vs. D (p = 0.0126), C vs. E (p < 0.0001), and C vs. F (p = 0.0050)."

- Line 216: what does "ultrasound history" mean? An individual who previously had an abdominal ultrasound? Some additional explanation would be helpful as it isn't clear.

Author’s response

There was an error in the original manuscript, and we apologize for the oversight. Line 216 has been corrected to read: “Regarding the history of UGIB, an increasing gradient of values was observed among patients with a history and a decreasing gradient for those without such a history, with statistical significance (p < 0.0001)”.

- Table 1: recommend using the term "range" instead of "variation" for age.

Author’s response

We thank the reviewer for this comment. The suggested modification has been implemented, and the term “range” has been used instead of “variation” for age in Table 1. The changes in Table 1 are highlighted in yellow in the manuscript.

- Table 2: does the fourth column list mean ELF values? It's not clear. If yes, please title the column as "Mean ELF". If the UGIB values are medians then I recommend putting a new column header just above the UGIB values. Currently, the footnotes are very confusing.

Author’s response

Indeed, Table 2 has been revised in the manuscript. The changes in Table 2 are highlighted in yellow in the manuscript.

- Figure 1: please state in the figure legend what the box and whiskers refer to (e.g., medians and IQRs?).

Author’s response

We thank the reviewer for the valuable comment. The figure legend has been updated to specify this information. These changes have been implemented in the manuscript.

The authors claim that using the ELF would be more useful and available in endemic areas due to the difficulty of obtaining an ultrasound. However, the three components of the ELF are not commonly available assays used in medical practice. Part of the Discussion should address how available the ELF really is in Sm-endemic areas of Brazil. Is this test truly more accessible than ultrasound, which despite requiring a trained operator, is mobile and doesn't require access to high-performance accredited laboratories. The justification for using ELF over ultrasound seems questionable.

Author’s response

Indeed, ELF is a patented test and is associated with relatively high costs. However, ultrasonography (US) also requires substantial investment, including the cost of equipment acquisition and additional expenses related to transporting the equipment to endemic areas. Furthermore, US has inherent operational costs, as it is an operator-dependent examination that requires the presence of a trained and experienced examiner in endemic regions to use the Niamey classification. It is also important to consider the logistical challenges associated with accessing many endemic areas. In this context, collecting blood samples in the field and transporting them to a specialized laboratory for ELF testing may represent a potentially more cost-effective strategy, despite the cost associated with the patented assay. Nevertheless, these costs are influenced by multiple logistical and operational factors and are therefore difficult to quantify objectively.

There are multiple grammatical and wording errors throughout the manuscript, including incorrect verb conjugations. A thorough editing by a fluent English speaker is highly recommended.
- Line 62: "samples" not "sample".

- Line 103: "eliminating" not "eliminate".

- Line 109: "develop" not "present".

- Line 115: "zones" not "zone".

- Lines 166-167: "by each participant" not "of each participant".

- Line 204: "Pearson" not "Person".

- Line 224: "ELF values" not "ELF value".

- Line 327: "the time" rather than "the case".

Author’s response

Thanks very much for this suggestion. We proofread the entire manuscript and corrected grammatical and typo errors, as well as improved fluency, in yellow.

Rebecca Dantas Thorp

Corresponding author
