# Supplementary material for: Evaluating periportal fibrosis in schistosomiasis mansoni using the ELF [Enhanced Liver Fibrosis] score: A pilot study
Source: PLoS Negl Trop Dis. 2026 Oct 9;20(10):e0014775. doi: 10.1371/journal.pntd.0014775 (PMC13671643; doi:10.1371/journal.pntd.0014775)
Supplement: Supplementary file 3 [file pntd.0014775.s004.doc]

**Ref: Major revision requested:** PNTD-D-25-01595R2**- [EMID:** 923c3748b0b41668**]**

Recife, August 16, 2026

**Dr. David J. Diemert, M.D**

**Academic Editor**

**PLOS Neglected Tropical Diseases**

Dear Dr. David J. Diemert, M.D,

We would like to express our sincere gratitude for the Editors and Reviewers for their thoughtful comments and constructive suggestions on our manuscript. We greatly appreciate the time and expertise devoted to its review. Their valuable feedback has helped us refine the manuscript, improve its clarity, and strengthen the presentation of our findings.

We are pleased to submit the revised version of our manuscript (PNTD-D-25-01595R1), entitled “Evaluating periportal fibrosis in schistosomiasis mansoni using the ELF [Enhanced Liver Fibrosis] score: a pilot study”. We hope that the revised version satisfactorily addresses the Reviewers’ concerns and is now suitable for publication.

To facilitate the review process, we have provided two files: A) "Tracked Changes" version: Showing all modifications made (yellow) to the original text. B) "Clean" version: With all changes accepted and formatted.

Below, we address the Reviewer's concern. Our responses are presented in Times New Roman font and blue color.

**Reviewer 2**

The Reviewer raised an important concern regarding the clarity of the table titles, particulary concerning the definition of the periportal fibrosis groups.

Tables should be better defined in the title ie what the Groups indicate.

Author’s response:

We thank the Reviewer for this important comment. We agree that the groups representing the different degrees of periportal fibrosis should be more clearly defined in the table titles. Accordingly, we have revised the relevant table titles to explicitly indicate the fibrosis patterns represented by each group according to the Niamey ultrasound classification: Group A+B (no fibrosis), Group C (mild fibrosis), Group D (moderate fibrosis), Group E (advanced fibrosis), and Group F (very advanced fibrosis). These changes were made to improve the clarity and interpretation of the tables.

Rebecca Dantas Thorp

Corresponding author
