# Supplementary material for: Evaluating periportal fibrosis in schistosomiasis mansoni using the ELF [Enhanced Liver Fibrosis] score: A pilot study
Source: PLoS Negl Trop Dis. 2026 Oct 9;20(10):e0014775. doi: 10.1371/journal.pntd.0014775 (PMC13671643; doi:10.1371/journal.pntd.0014775)
Supplement: Peer review history

# Peer review: Decision Letter 0

**DOI:** 10.1371/journal.pntd.0014775.r001

**Authors:** David Diemert, Gabriel Rinaldi

23 Dec 2025

PNTD-D-25-01595

Evaluation of periportal fibrosis in schistosomiasis mansoni using the ELF [Enhanced Liver Fibrosis] score

PLOS Neglected Tropical Diseases

Dear Dr. Thorp,

Thank you for submitting your manuscript to PLOS Neglected Tropical Diseases. After careful consideration, we feel that it has merit but does not fully meet PLOS Neglected Tropical Diseases's publication criteria as it currently stands. Therefore, we invite you to submit a revised version of the manuscript that addresses the points raised during the review process.

Please submit your revised manuscript within by Feb 21 2026 11:59PM. If you will need more time than this to complete your revisions, please reply to this message or contact the journal office at plosntds@plos.org. When you're ready to submit your revision, log on to https://www.editorialmanager.com/pntd/ and select the 'Submissions Needing Revision' folder to locate your manuscript file.

Please include the following items when submitting your revised manuscript:

* A letter that responds to each point raised by the editor and reviewer(s). You should upload this letter as a separate file labeled 'Response to Reviewers'. This file does not need to include responses to any formatting updates and technical items listed in the 'Journal Requirements' section below.

* A marked-up copy of your manuscript that highlights changes made to the original version. You should upload this as a separate file labeled 'Revised Manuscript with Track Changes'.

* An unmarked version of your revised paper without tracked changes. You should upload this as a separate file labeled 'Manuscript'.

If you would like to make changes to your financial disclosure, competing interests statement, or data availability statement, please make these updates within the submission form at the time of resubmission. Guidelines for resubmitting your figure files are available below the reviewer comments at the end of this letter.

We look forward to receiving your revised manuscript.

Kind regards,

David J. Diemert, M.D.

Academic Editor

PLOS Neglected Tropical Diseases

Gabriel Rinaldi

Section Editor

PLOS Neglected Tropical Diseases

Shaden Kamhawi

co-Editor-in-Chief

PLOS Neglected Tropical Diseases

orcid.org/0000-0003-4304-636XX

Paul Brindley

co-Editor-in-Chief

PLOS Neglected Tropical Diseases

orcid.org/0000-0003-1765-0002

**Journal Requirements:**

1) Please ensure that the CRediT author contributions listed for every co-author are completed accurately and in full.

At this stage, the following Authors/Authors require contributions: Rebecca Dantas Thorp, Andrea Dória Batista, Carolline Araújo Mariz, Lilian Rose Maia Gomes de Araújo, George Tadeu Nunes Diniz, Ana Virgínia Matos Sá Barreto, Clarice Neueschwander Lins de Morais, Paula Carolina Valença Silva, Ana Lúcia Coutinho Domingues, and Edmundo Pessoa Lopes. Please ensure that the full contributions of each author are acknowledged in the "Add/Edit/Remove Authors" section of our submission form.

The list of CRediT author contributions may be found here: https://journals.plos.org/plosntds/s/authorship#loc-author-contributions

2) We ask that a manuscript source file is provided at Revision. Please upload your manuscript file as a .doc, .docx, .rtf or .tex. If you are providing a .tex file, please upload it under the item type u2018LaTeX Source Fileu2019 and leave your .pdf version as the item type u2018Manuscriptu2019.

3) Please upload all main figures as separate Figure files in .tif or .eps format. For more information about how to convert and format your figure files please see our guidelines:

https://journals.plos.org/plosntds/s/figures

4) We have noticed that you have a list of Supporting Information legends in your manuscript. However, there are no corresponding files uploaded to the submission. Please upload them as separate files with the item type 'Supporting Information'.

**Reviewers' Comments:**

Reviewer's Responses to Questions

**Key Review Criteria Required for Acceptance?**

As you describe the new analyses required for acceptance, please consider the following:

**Methods**

-Are the objectives of the study clearly articulated with a clear testable hypothesis stated?

-Is the study design appropriate to address the stated objectives?

-Is the population clearly described and appropriate for the hypothesis being tested?

-Is the sample size sufficient to ensure adequate power to address the hypothesis being tested?

-Were correct statistical analysis used to support conclusions?

-Are there concerns about ethical or regulatory requirements being met?

Reviewer #1: (No Response)

**********

**Results**

-Does the analysis presented match the analysis plan?

-Are the results clearly and completely presented?

-Are the figures (Tables, Images) of sufficient quality for clarity?

Reviewer #1: (No Response)

**********

**Conclusions**

-Are the conclusions supported by the data presented?

-Are the limitations of analysis clearly described?

-Do the authors discuss how these data can be helpful to advance our understanding of the topic under study?

-Is public health relevance addressed?

Reviewer #1: (No Response)

**********

**Editorial and Data Presentation Modifications?**

Use this section for editorial suggestions as well as relatively minor modifications of existing data that would enhance clarity. If the only modifications needed are minor and/or editorial, you may wish to recommend “Minor Revision” or “Accept”.

Reviewer #1: (No Response)

**********

**Summary and General Comments**

Use this section to provide overall comments, discuss strengths/weaknesses of the study, novelty, significance, general execution and scholarship. You may also include additional comments for the author, including concerns about dual publication, research ethics, or publication ethics. If requesting major revision, please articulate the new experiments that are needed.

Reviewer #1: This paper presents a small study in a Brasilian schistosomiasis clinic where 196 patients were studied, and a new biomarker was measured in attempts of associating with image-based patterns. The study lacks a comparison to other biomarkers, inadequate information to follow the study design (sampling justification, controlling for co-infections, justification for image pattern groupings, or quality control for image diagnoses). The discussion would require major rewriting to focus only on comparisons that are relevant to the data actually analyzed in this study. I suggest the title be rewritten to indicate this is a pilot study of ELF measurements in schistosomiasis patients.

Introduction

- Line 70: What is meant by serious infections? Define. Again, what is a severe infection? Line 74. Use exact terminology of infection intensities.

- The introduction should be rewritten to focus on the different indicators of fibrosis and a direct comparison, including citing studies that have shown their past agreement. A recent review, in their data extraction table, note all studies performing both biomarker analyses and imaging and their agreement. https://journals.plos.org/plosntds/article?id=10.1371/journal.pntd.0012033

- Biological indicators are not necessarily better, given they are reliant on the time point of the sample, vary widely, and are non-specific. Please indicate these limitations. Also, give examples of associations (strength, direction, population studied) of the indices quoted from past studies (Lines 95-96).

- Line 100: Issues in acknowledging a gold/reference standard for fibrosis are needed to be acknowledged. Niamey is by no means a gold standard.

Methods

- How were patients sampled? What was the selection strategy? Provide information on the sampling register choice, strategy, and power calculations.

- Give the frequency range of the transducer.

- Was ultrasound performed by a sonographer who knew the ELF results? If so, this setup is a limitation and could have biased the sonographer’s image results. It should be acknowledged in the discussion.

- Was any re-reading of the ultrasound images done to assess the quality of the diagnosis? If not, acknowledge as a limitation.

- Were any of the distributions of ELF normal? If not, just describe the statistical test actually used.

- Do you mean chi2 was used for categorical data? When was fisher’s exact p-value calculated? Be specific.

- Indicate R version.

- Why wasn’t ELF compared to other biomarkers or indices?

- ELF needs to be better introduced, explaining it’s function and biological measurement.

Results

- What is the p-value here comparing?! Lines 170-173. Give values of both comparison groups. It doesn’t make sense to put p-values on simple descriptives of prevalence unless something is being compared. Please write out the comparison groups.

- Table 2: Show the standard deviations not the min and max values. We need to know what is being compared. Also, why does it say mean in the column header and median in the footnote? What is being compared?? If median then show the median and the interquartile range. If mean then show mean and standard deviation. It is not clear the right statistical tests were used. We also need to see the observations/patients in each category compared.

- Fig. 1 why are there no differences between a+b vs c? Why is a+b higher than c?

- A, which represents normal should be analyzed separately. This would allow answering the question as to whether ELF differentiates between normal and fibrosed. Then the next question is whether it differentiates patterns associated to periportal fibrosis.

- Fig 2. Where are the regression models for these results??

Discussion

- Line 256: Why are you discussing fatty livers here and you haven’t presented any data in this are?

- What is being claimed that ELF is associated with? Many disconnected examples are provided, but it does not appear that this study actually looked at co-morbidities or co-infections, so we have no real idea if it is distinguishing schistosomal liver fibrosis.

- It is a limitation that this study has not been compared to other biomarkers.

Minor

- The manuscript needs to be read for grammatical errors, typos, and fluency in language.

- Avoid paragraphs of fewer than three sentences.

- Spell out all abbreviations on first usage.

- Shorten Table, etc titles.

- Upper GI bleeding = UGIB not UGB.

**********

PLOS authors have the option to publish the peer review history of their article (what does this mean?). If published, this will include your full peer review and any attached files.

If you choose “no”, your identity will remain anonymous but your review may still be made public.

**Do you want your identity to be public for this peer review?**  For information about this choice, including consent withdrawal, please see our Privacy Policy.

Reviewer #1: No

[NOTE: If reviewer comments were submitted as an attachment file, they will be attached to this email and accessible via the submission site. Please log into your account, locate the manuscript record, and check for the action link "View Attachments". If this link does not appear, there are no attachment files.]

**Figure resubmission:**  While revising your submission, we strongly recommend that you use PLOS’s NAAS tool (https://ngplosjournals.pagemajik.ai/artanalysis) to test your figure files. NAAS can convert your figure files to the TIFF file type and meet basic requirements (such as print size, resolution), or provide you with a report on issues that do not meet our requirements and that NAAS cannot fix.

After uploading your figures to PLOS’s NAAS tool - https://ngplosjournals.pagemajik.ai/artanalysis, NAAS will process the files provided and display the results in the "Uploaded Files" section of the page as the processing is complete. If the uploaded figures meet our requirements (or NAAS is able to fix the files to meet our requirements), the figure will be marked as "fixed" above. If NAAS is unable to fix the files, a red "failed" label will appear above. When NAAS has confirmed that the figure files meet our requirements, please download the file via the download option, and include these NAAS processed figure files when submitting your revised manuscript.

**Reproducibility:**

To enhance the reproducibility of your results, we recommend that authors of applicable studies deposit laboratory protocols in protocols.io, where a protocol can be assigned its own identifier (DOI) such that it can be cited independently in the future. Additionally, PLOS ONE offers an option to publish peer-reviewed clinical study protocols. Read more information on sharing protocols at https://plos.org/protocols?utm_medium=editorial-email&utm_source=authorletters&utm_campaign=protocols

# Author comment: Author response to Decision Letter 1

**DOI:** 10.1371/journal.pntd.0014775.r002

23 Feb 2026

Attachment Submitted filename: Response to Reviewers.doc

# Peer review: Decision Letter 1

**DOI:** 10.1371/journal.pntd.0014775.r003

**Authors:** Gabriel Rinaldi

5 Jun 2026

PNTD-D-25-01595R1

Evaluating periportal fibrosis in schistosomiasis mansoni using the ELF [Enhanced Liver Fibrosis] score: a pilot study

PLOS Neglected Tropical Diseases

Dear Dr. Thorp,

Thank you for submitting your manuscript to PLOS Neglected Tropical Diseases. After careful consideration, we feel that it has merit but does not fully meet PLOS Neglected Tropical Diseases's publication criteria as it currently stands. Therefore, we invite you to submit a revised version of the manuscript that addresses the points raised during the review process.

Please submit your revised manuscript within by Jul 05 2026 11:59PM. If you will need more time than this to complete your revisions, please reply to this message or contact the journal office at plosntds@plos.org. When you're ready to submit your revision, log on to https://www.editorialmanager.com/pntd/ and select the 'Submissions Needing Revision' folder to locate your manuscript file.

Please include the following items when submitting your revised manuscript:

* A letter that responds to each point raised by the editor and reviewer(s). You should upload this letter as a separate file labeled 'Response to Reviewers'. This file does not need to include responses to any formatting updates and technical items listed in the 'Journal Requirements' section below.

* A marked-up copy of your manuscript that highlights changes made to the original version. You should upload this as a separate file labeled 'Revised Manuscript with Track Changes'.

* An unmarked version of your revised paper without tracked changes. You should upload this as a separate file labeled 'Manuscript'.

If you would like to make changes to your financial disclosure, competing interests statement, or data availability statement, please make these updates within the submission form at the time of resubmission. Guidelines for resubmitting your figure files are available below the reviewer comments at the end of this letter.

As the corresponding author, your ORCID iD is verified in the submission system and will appear in the published article. PLOS supports the use of ORCID, and we encourage all coauthors to register for an ORCID iD and use it as well. Please encourage your coauthors to verify their ORCID iD within the submission system before final acceptance, as unverified ORCID iDs will not appear in the published article. Only the individual author can complete the verification step; PLOS staff cannot verify ORCID iDs on behalf of authors.

We look forward to receiving your revised manuscript.

Kind regards,

Gabriel Rinaldi, M.D., Ph.D.

Section Editor

PLOS Neglected Tropical Diseases

Gabriel Rinaldi

Section Editor

PLOS Neglected Tropical Diseases

Shaden Kamhawi

co-Editor-in-Chief

PLOS Neglected Tropical Diseases

orcid.org/0000-0003-4304-636XX

Paul Brindley

co-Editor-in-Chief

PLOS Neglected Tropical Diseases

orcid.org/0000-0003-1765-0002

**Additional Editor Comments (if provided):**

The reviewers have raised several important concerns regarding this study that require a comprehensive response from the authors. In particular, one reviewer identified potential overlap between the present manuscript and previously reported work, raising questions about the novelty of the study, the relationship between the datasets, and the authorship of related publications.

Please provide a detailed point-by-point clarification addressing all issues raised by the reviewers. Special attention should be given to the following comment:

“Why was this 2017 study not referenced and compared: Diagnosing schistosomiasis-induced liver morbidity: implications for global control? The Ozaki et al. (2024) study is presented only as a conference abstract (A-250 Evaluation of Liver Fibrosis in Schistosomiasis Mansoni Using the ELF [Enhanced Liver Fibrosis] Score). Why is Ozaki not listed as an author on the present publication or on the related abstract available at: https://www.apm.org.br/wp-content/uploads/SPMJ_v142Suppl1.pdf? Given the differences in authorship across these reports, it is unclear who was involved in the study and how the various datasets and publications are related.”

The authors should clearly explain the relationship between the current manuscript and any previously published articles, abstracts, conference presentations, or related reports, including whether the same study population, dataset, or analyses were used. In addition, the authors should clarify the contributions of all individuals involved and address any apparent discrepancies in authorship across related publications.

**Journal Requirements:**

If the reviewer comments include a recommendation to cite specific previously published works, please review and evaluate these publications to determine whether they are relevant and should be cited. There is no requirement to cite these works unless the editor has indicated otherwise.

1) We note that your Data Availability Statement is currently as follows: "We confirm that all data reported in the manuscript are publicly available. The authors are making all reported data and metadata related to the study results available, as provided in the submission. All data can be found in the results section of the manuscript, as well as in the Supporting Information section.". Please confirm at this time whether or not your submission contains all raw data required to replicate the results of your study. Authors must share the “minimal data set” for their submission. PLOS defines the minimal data set to consist of the data required to replicate all study findings reported in the article, as well as related metadata and methods (https://journals.plos.org/plosone/s/data-availability#loc-minimal-data-set-definition).

For example, authors should submit the following data:

1) The values behind the means, standard deviations and other measures reported;

2) The values used to build graphs;

3) The points extracted from images for analysis..

Authors do not need to submit their entire data set if only a portion of the data was used in the reported study.

If your submission does not contain these data, please either upload them as Supporting Information files or deposit them to a stable, public repository and provide us with the relevant URLs, DOIs, or accession numbers. For a list of recommended repositories, please see https://journals.plos.org/plosone/s/recommended-repositories.

If there are ethical or legal restrictions on sharing a de-identified data set, please explain them in detail (e.g., data contain potentially sensitive information, data are owned by a third-party organization, etc.) and who has imposed them (e.g., an ethics committee). Please also provide contact information for a data access committee, ethics committee, or other institutional body to which data requests may be sent. If data are owned by a third party, please indicate how others may request data access.

**Reviewers' Comments:**

Reviewer's Responses to Questions

**Key Review Criteria Required for Acceptance?**

As you describe the new analyses required for acceptance, please consider the following:

**Methods**

-Are the objectives of the study clearly articulated with a clear testable hypothesis stated?

-Is the study design appropriate to address the stated objectives?

-Is the population clearly described and appropriate for the hypothesis being tested?

-Is the sample size sufficient to ensure adequate power to address the hypothesis being tested?

-Were correct statistical analysis used to support conclusions?

-Are there concerns about ethical or regulatory requirements being met?

Reviewer #2: (No Response)

Reviewer #3: -The ultrasound PPS scoring system (A-F) needs to be better explained in the Methods. In the Results section, it becomes very confusing when the authors alternately refer to "no PPS", "mild or moderate PPS", vs. the letter designations. For this reviewer who is not an expert on ultrasound or portal hypertension, it took several reads to understand that grades A+B are equivalent to "no PPS" (I think). The severity definitions of each letter grade need to be spelled out.

- Lines 168-169: it appears that the participants were classified as having had Sm based only on history of prior contact with water or treatment with praziquantel, without any confirmation of diagnosis with a parasitologic test (e.g., stool microscopy and/or serology). This likely resulted in inclusion of individuals who did not in fact have Sm. The authors should comment on this limitation for bias being introduced to the results based on the definition of Sm infection.

**********

**Results**

-Does the analysis presented match the analysis plan?

-Are the results clearly and completely presented?

-Are the figures (Tables, Images) of sufficient quality for clarity?

Reviewer #2: (No Response)

Reviewer #3: - It is unclear why Table 2 needs 4 panels since it's looking at the same data just grouped together into different bins. Nothing more is added by panels C and D that isn't already evident from panels A and B.

- At no point in the Results is it pointed out that the ELF values in the Niamey C group were actually lower than A+B. It is a bit misleading to state that these two groups were significantly different when in fact the difference goes against the main finding that increasing ELF scores correlate with increasing PPS on ultrasound. The discrepancy between A+B and C needs to be clarified.

- Why do the authors present mean ELF scores in Table 1 but use medians for Figure 2? It makes no sense to use different measures.

- Lines 213-215: it would be helpful not just to say that there was a statistically significant difference in age between the PPF levels, but that increasing PPF score was associated with increasing age (in general).

- Line 216: what does "ultrasound history" mean? An individual who previously had an abdominal ultrasound? Some additional explanation would be helpful as it isn't clear.

- Table 1: recommend using the term "range" instead of "variation" for age.

- Table 2: does the fourth column list mean ELF values? It's not clear. If yes, please title the column as "Mean ELF". If the UGIB values are medians then I recommend putting a new column header just above the UGIB values. Currently, the footnotes are very confusing.

- Figure 1: please state in the figure legend what the box and whiskers refer to (e.g., medians and IQRs?).

**********

**Conclusions**

-Are the conclusions supported by the data presented?

-Are the limitations of analysis clearly described?

-Do the authors discuss how these data can be helpful to advance our understanding of the topic under study?

-Is public health relevance addressed?

Reviewer #2: (No Response)

Reviewer #3: - The authors claim that using the ELF would be more useful and available in endemic areas due to the difficulty of obtaining an ultrasound. However, the three components of the ELF are not commonly available assays used in medical practice. Part of the Discussion should address how available the ELF really is in Sm-endemic areas of Brazil. Is this test truly more accessible than ultrasound, which despite requiring a trained operator, is mobile and doesn't require access to high-performance accredited laboratories. The justification for using ELF over ultrasound seems questionable.

**********

**Editorial and Data Presentation Modifications?**

Use this section for editorial suggestions as well as relatively minor modifications of existing data that would enhance clarity. If the only modifications needed are minor and/or editorial, you may wish to recommend “Minor Revision” or “Accept”.

Reviewer #2: (No Response)

Reviewer #3: - There are multiple grammatical and wording errors throughout the manuscript, including incorrect verb conjugations. A thorough editing by a fluent English speaker is highly recommended.

Some examples:

- Line 62: "samples" not "sample".

- Line 103: "eliminating" not "eliminate".

- Line 109: "develop" not "present".

- Line 115: "zones" not "zone".

- Lines 166-167: "by each participant" not "of each participant".

- Line 204: "Pearson" not "Person".

- Line 224: "ELF values" not "ELF value".

- Line 327: "the time" rather than "the case".

**********

**Summary and General Comments**

Use this section to provide overall comments, discuss strengths/weaknesses of the study, novelty, significance, general execution and scholarship. You may also include additional comments for the author, including concerns about dual publication, research ethics, or publication ethics. If requesting major revision, please articulate the new experiments that are needed.

Reviewer #2: This is a straight forward but interesting study. I was not involved with the first review.

Some lack of literature analysis appears to be the case.

Why was this 2017 study not reference and compared “Diagnosing schistosomiasis-induced liver morbidity: implications for global control”

The ozaki paper 2024 study is a conference abstract only “A-250 Evaluation of Liver Fibrosis in Schistosomiasis Mansoni Using the ELF (Enhanced Liver Fibrosis) Score”, why is Ozaki not on this publication

Or here too?

https://www.apm.org.br/wp-content/uploads/SPMJ_v142Suppl1.pdf

Again with different authors, so it is not clear who is actually involved with this study.

Reviewer #3: (No Response)

**********

PLOS authors have the option to publish the peer review history of their article (what does this mean?). If published, this will include your full peer review and any attached files.

If you choose “no”, your identity will remain anonymous but your review may still be made public.

**Do you want your identity to be public for this peer review?**  For information about this choice, including consent withdrawal, please see our Privacy Policy.

Reviewer #2: No

Reviewer #3: No

[NOTE: If reviewer comments were submitted as an attachment file, they will be attached to this email and accessible via the submission site. Please log into your account, locate the manuscript record, and check for the action link "View Attachments". If this link does not appear, there are no attachment files.]

**Figure resubmission:**

While revising your submission, we strongly recommend that you use PLOS’s NAAS tool (https://ngplosjournals.pagemajik.ai/artanalysis) to test your figure files. NAAS can convert your figure files to the TIFF file type and meet basic requirements (such as print size, resolution), or provide you with a report on issues that do not meet our requirements and that NAAS cannot fix.

After uploading your figures to PLOS’s NAAS tool - https://ngplosjournals.pagemajik.ai/artanalysis, NAAS will process the files provided and display the results in the "Uploaded Files" section of the page as the processing is complete. If the uploaded figures meet our requirements (or NAAS is able to fix the files to meet our requirements), the figure will be marked as "fixed" above. If NAAS is unable to fix the files, a red "failed" label will appear above. When NAAS has confirmed that the figure files meet our requirements, please download the file via the download option, and include these NAAS processed figure files when submitting your revised manuscript.

**Reproducibility:**

To enhance the reproducibility of your results, we recommend that authors of applicable studies deposit laboratory protocols in protocols.io, where a protocol can be assigned its own identifier (DOI) such that it can be cited independently in the future. Additionally, PLOS ONE offers an option to publish peer-reviewed clinical study protocols. Read more information on sharing protocols at https://plos.org/protocols?utm_medium=editorial-email&utm_source=authorletters&utm_campaign=protocols

# Author comment: Author response to Decision Letter 2

**DOI:** 10.1371/journal.pntd.0014775.r004

8 Jul 2026

Attachment Submitted filename: Response_to_Reviewers_auresp_2.doc

# Peer review: Decision Letter 2

**DOI:** 10.1371/journal.pntd.0014775.r005

**Authors:** Gabriel Rinaldi

7 Aug 2026

PNTD-D-25-01595R2Evaluating periportal fibrosis in schistosomiasis mansoni using the ELF [Enhanced Liver Fibrosis] score: a pilot studyPLOS Neglected Tropical Diseases Dear Dr. Thorp, Thank you for submitting your manuscript to PLOS Neglected Tropical Diseases. After careful consideration, we feel that it has merit but does not fully meet PLOS Neglected Tropical Diseases's publication criteria as it currently stands. Therefore, we invite you to submit a revised version of the manuscript that addresses the points raised during the review process. Please submit your revised manuscript by Sep 06 2026 11:59PM. If you will need more time than this to complete your revisions, please reply to this message or contact the journal office at plosntds@plos.org. When you're ready to submit your revision, log on to https://www.editorialmanager.com/pntd/ and select the 'Submissions Needing Revision' folder to locate your manuscript file. Please include the following items when submitting your revised manuscript:* A letter that responds to each point raised by the editor and reviewer(s). You should upload this letter as a separate file labeled 'Response to Reviewers'. This file does not need to include responses to any formatting updates and technical items listed in the 'Journal Requirements' section below.* A marked-up copy of your manuscript that highlights changes made to the original version. You should upload this as a separate file labeled 'Revised Manuscript with Track Changes'.* An unmarked version of your revised paper without tracked changes. You should upload this as a separate file labeled 'Manuscript'. If you would like to make changes to your financial disclosure, competing interests statement, or data availability statement, please make these updates within the submission form at the time of resubmission. Guidelines for resubmitting your figure files are available below the reviewer comments at the end of this letter. As the corresponding author, your ORCID iD is verified in the submission system and will appear in the published article. PLOS supports the use of ORCID, and we encourage all coauthors to register for an ORCID iD and use it as well. Please encourage your coauthors to verify their ORCID iD within the submission system before final acceptance, as unverified ORCID iDs will not appear in the published article. Only the individual author can complete the verification step; PLOS staff cannot verify ORCID iDs on behalf of authors.We look forward to receiving your revised manuscript. Kind regards, Gabriel Rinaldi, M.D., Ph.D.Section EditorPLOS Neglected Tropical Diseases Gabriel RinaldiSection EditorPLOS Neglected Tropical Diseases

Shaden Kamhawi

co-Editor-in-Chief

PLOS Neglected Tropical Diseases

orcid.org/0000-0003-4304-636XX

Paul Brindley

co-Editor-in-Chief

PLOS Neglected Tropical Diseases

orcid.org/0000-0003-1765-0002

**Reviewers' comments:**  Reviewer's Responses to Questions

**Key Review Criteria Required for Acceptance?**

As you describe the new analyses required for acceptance, please consider the following:

**Methods**

-Are the objectives of the study clearly articulated with a clear testable hypothesis stated?

-Is the study design appropriate to address the stated objectives?

-Is the population clearly described and appropriate for the hypothesis being tested?

-Is the sample size sufficient to ensure adequate power to address the hypothesis being tested?

-Were correct statistical analysis used to support conclusions?

-Are there concerns about ethical or regulatory requirements being met?

Reviewer #2: (No Response)

Reviewer #3: (No Response)

**********

**Results**

-Does the analysis presented match the analysis plan?

-Are the results clearly and completely presented?

-Are the figures (Tables, Images) of sufficient quality for clarity?

Reviewer #2: Tables should be better defined in the title ie what the Groups indicate.

Reviewer #3: (No Response)

**********

**Conclusions**

-Are the conclusions supported by the data presented?

-Are the limitations of analysis clearly described?

-Do the authors discuss how these data can be helpful to advance our understanding of the topic under study?

-Is public health relevance addressed?

Reviewer #2: -

Reviewer #3: (No Response)

**********

**Editorial and Data Presentation Modifications?**

Use this section for editorial suggestions as well as relatively minor modifications of existing data that would enhance clarity. If the only modifications needed are minor and/or editorial, you may wish to recommend “Minor Revision” or “Accept”.

Reviewer #2: -

Reviewer #3: (No Response)

**********

**Summary and General Comments**

Use this section to provide overall comments, discuss strengths/weaknesses of the study, novelty, significance, general execution and scholarship. You may also include additional comments for the author, including concerns about dual publication, research ethics, or publication ethics. If requesting major revision, please articulate the new experiments that are needed.

Reviewer #2: -

Reviewer #3: The authors have responded acceptably to the prior reviews.

**********

PLOS authors have the option to publish the peer review history of their article (what does this mean?). If published, this will include your full peer review and any attached files.

If you choose “no”, your identity will remain anonymous but your review may still be made public.

**Do you want your identity to be public for this peer review?**  For information about this choice, including consent withdrawal, please see our Privacy Policy.

Reviewer #2: No

Reviewer #3: No

[NOTE: If reviewer comments were submitted as an attachment file, they will be attached to this email and accessible via the submission site. Please log into your account, locate the manuscript record, and check for the action link "View Attachments". If this link does not appear, there are no attachment files.] **Figure resubmission:**  While revising your submission, we strongly recommend that you use PLOS’s NAAS tool (https://ngplosjournals.pagemajik.ai/artanalysis) to test your figure files. NAAS can convert your figure files to the TIFF file type and meet basic requirements (such as print size, resolution), or provide you with a report on issues that do not meet our requirements and that NAAS cannot fix.

After uploading your figures to PLOS’s NAAS tool - https://ngplosjournals.pagemajik.ai/artanalysis, NAAS will process the files provided and display the results in the "Uploaded Files" section of the page as the processing is complete. If the uploaded figures meet our requirements (or NAAS is able to fix the files to meet our requirements), the figure will be marked as "fixed" above. If NAAS is unable to fix the files, a red "failed" label will appear above. When NAAS has confirmed that the figure files meet our requirements, please download the file via the download option, and include these NAAS processed figure files when submitting your revised manuscript. **Reproducibility:**  To enhance the reproducibility of your results, we recommend that authors of applicable studies deposit laboratory protocols in protocols.io, where a protocol can be assigned its own identifier (DOI) such that it can be cited independently in the future. Additionally, PLOS ONE offers an option to publish peer-reviewed clinical study protocols. Read more information on sharing protocols at https://plos.org/protocols?utm_medium=editorial-email&utm_source=authorletters&utm_campaign=protocols

# Author comment: Author response to Decision Letter 3

**DOI:** 10.1371/journal.pntd.0014775.r006

31 Aug 2026

Attachment Submitted filename: Response_to_Reviewers_auresp_3.doc

# Editor report: Decision Letter 3

**DOI:** 10.1371/journal.pntd.0014775.r007

**Authors:** Gabriel Rinaldi

21 Sep 2026

Dear Thorp,

We are pleased to inform you that your manuscript 'Evaluating periportal fibrosis in schistosomiasis mansoni using the ELF [Enhanced Liver Fibrosis] score: a pilot study' has been provisionally accepted for publication in PLOS Neglected Tropical Diseases.

Before your manuscript can be formally accepted you will need to complete some formatting changes, which you will receive in a follow up email. A member of our team will be in touch with a set of requests.

Please note that your manuscript will not be scheduled for publication until you have made the required changes, so a swift response is appreciated.

IMPORTANT: The editorial review process is now complete. PLOS will only permit corrections to spelling, formatting or significant scientific errors from this point onwards. Requests for major changes, or any which affect the scientific understanding of your work, will cause delays to the publication date of your manuscript.

Should you, your institution's press office or the journal office choose to press release your paper, you will automatically be opted out of early publication. We ask that you notify us now if you or your institution is planning to press release the article. All press must be co-ordinated with PLOS.

Thank you again for supporting Open Access publishing; we are looking forward to publishing your work in PLOS Neglected Tropical Diseases.

Best regards,

Gabriel Rinaldi, M.D., Ph.D.

Section Editor

PLOS Neglected Tropical Diseases

Gabriel Rinaldi

Section Editor

PLOS Neglected Tropical Diseases

Shaden Kamhawi

co-Editor-in-Chief

PLOS Neglected Tropical Diseases

orcid.org/0000-0003-4304-636XX

Paul Brindley

co-Editor-in-Chief

PLOS Neglected Tropical Diseases

orcid.org/0000-0003-1765-0002

***********************************************************

# Editor report: Acceptance letter

**DOI:** 10.1371/journal.pntd.0014775.r008

**Authors:** Gabriel Rinaldi

Dear Thorp,

We are delighted to inform you that your manuscript, "Evaluating periportal fibrosis in schistosomiasis mansoni using the ELF [Enhanced Liver Fibrosis] score: a pilot study," has been formally accepted for publication in PLOS Neglected Tropical Diseases.

We have now passed your article onto the PLOS Production Department who will complete the rest of the publication process. All authors will receive a confirmation email upon publication.

The corresponding author will soon be receiving a typeset proof for review, to ensure errors have not been introduced during production. Please review the PDF proof of your manuscript carefully, as this is the last chance to correct any scientific or type-setting errors. Please note that major changes, or those which affect the scientific understanding of the work, will likely cause delays to the publication date of your manuscript. Note: Proofs for Front Matter articles (Editorial, Viewpoint, Symposium, Review, etc...) are generated on a different schedule and may not be made available as quickly.

Soon after your final files are uploaded, the early version of your manuscript will be published online unless you opted out of this process. The date of the early version will be your article's publication date. The final article will be published to the same URL, and all versions of the paper will be accessible to readers.

For Research Articles, you will receive an invoice from PLOS for your publication fee after your manuscript has reached the completed accept phase. If you receive an email requesting payment before acceptance or for any other service, this may be a phishing scheme. Learn how to identify phishing emails and protect your accounts at https://explore.plos.org/phishing.

Thank you again for supporting open-access publishing; we are looking forward to publishing your work in PLOS Neglected Tropical Diseases.

Best regards,

Shaden Kamhawi

co-Editor-in-Chief

PLOS Neglected Tropical Diseases

Paul Brindley

co-Editor-in-Chief

PLOS Neglected Tropical Diseases
